# Construction of Efficient High-Rate Protograph QC-LDPC Codes by Joint EXIT Chart, PEG, AWD, and QC-NLACE Techniques

**DOI:** 10.3390/e28060604

**Published:** 2026-05-28

**Authors:** Ying Chen, Jianrong Bao, Yanhai Shang, Chao Liu, Shenji Luan

**Affiliations:** 1 Information Engineering College, Hangzhou Dianzi University, Hangzhou 311305, China; cheny@hdu.edu.cn (Y.C.); yhshang@hdu.edu.cn (Y.S.); luanshenji@hdu.edu.cn (S.L.); 2 School of Communication Engineering, Hangzhou Dianzi University, Hangzhou 310018, China; baojr@hdu.edu.cn; 3 National Mobile Communications Research Laboratory, Southeast University, Nanjing 210096, China

**Keywords:** protograph QC-LDPC codes, extrinsic information transfer chart, progressive-edge-growth, asymptotic weight distribution, nested loop approximated cycle extrinsic message degree

## Abstract

To obtain efficient channel codes with high power efficiency at moderate signal-to-noise ratios (SNRs), an efficient high-rate protograph quasi-cyclic (QC) low-density parity-check (LDPC) codes is optimally constructed. By an optimized protograph template, the code framework is firstly produced by the extensions of the variable nodes. By enlarging the dimension of the sub-matrices related to the protograph framework, the base QC matrix template is generated with required code rate and length by the extrinsic information transfer (EXIT) chart for better decoding threshold. Then, the elements in the base matrix are split with even smaller square sub-matrices of the same row and column weights. In this procedure, a progressive-edge-growth (PEG) algorithm is employed to find the optimized positions of the QC sub-matrices to obtain larger girth for better error floor performance. Moreover, an asymptotic weight distribution (AWD) is employed to keep a low-code-error floor for the code. Also the circulant offsets in all QC sub-matrices are optimally searched by a QC oriented nested loop approximated cycle extrinsic message degree (QC-NLACE) algorithm, which improves the relationship of the unavoidable loops in the code’s Tanner graph to cut the error floor. Simulation results show that the codes produced by the proposed method show quite good bit-error-rate (BER) performance. In addition, they exhibit good properties of high spectrum efficiency brought by the high code rate, and the low complexity by the short code length. Moreover, a series of different rate-compatible LDPC codes can be generated from the same protograph framework with some variable node extensions, which significantly eases the code design. Therefore, the proposed code construction can be efficiently applied in the optimal construction of high-rate and short-length rate-compatible QC-LDPC codes with a high data rate and rational complexity, which makes the codes extremely suited for use in new-generation power-constrained wireless communications.

## 1. Introduction

Nowadays, the channel coding demand for mobile Internet and the Internet of things poses a challenging requirement for 5th generation (5G) wireless communications [1]. They required good channel codes for high throughput, low power dissipation, and low latency. LDPC codes, originally proposed by Gallager in 1962, were rediscovered by Mackay in 1995 [2]. They had received extensive attention because of their ability to approach Shannon capacity and parallel decoding. Initially, they were constructed with random check matrices, the random LDPC codes, to have dense generator matrices of high encoding complexity of the square of code length. Random LDPC codes, as a result of carefully chosen degree profiles, achieved Shannon capacity at a close rate, when decoded by the belief propagation (BP) algorithm [3]. However, they still employed irregular and complex encoders to hinder their practice. So, it was necessary to construct structured LDPC codes, e.g., repeat accumulate (RA) codes [4] and quasi-cyclic (QC) LDPC codes [5], for efficient encoding and a low error floor. High-rate LDPC codes were applied in digital wireless communications with especially high spectrum efficiency. The LDPC codes can be applied in a system characterized by highly limited computational resources. And the classic Richardson–Urbanke algorithm can be decomposed for the QC-LDPC subclass into cyclic shifts and GF(2) additions, directly corresponding to the CPU instructions [6]. But short, high-rate QC LDPC codes were hard to be constructed with both low floors and good thresholds [7]. In addition, the efficiency and low-complexity encoding with the structure was extensively studied in the implementation of the LDPC encoder and decoder. It was mainly derived from the standard by the recursive iterative encoding algorithms, specifically targeting high computational encoding complexity [8]. For decoding, a novel base graph with static scheduling was proposed for layered decoding of 5G LDPC codes to accelerate decoding convergence and reduce the average iterations [9]. Therefore, it was necessary to investigate structured, high-rate QC-LDPC codes with easily encodable structure for high spectrum efficiency and low complexity in the variable wireless channels.

LDPC codes are presented by Tanner graphs for concise coding analyses. The variable and check nodes in a Tanner graph were mapped to the code words and the parity-check relationships [2]. Recently, low-rate, structured LDPC codes, i.e., accumulate-repeat-accumulate (ARA) codes, were presented with low floor and good threshold [10]. They were produced by a protograph [11] for good performance. The distortion rate of these protograph-like LDPC codes for Bernoulli sources over AWGN channels was investigated, where the the additional distortion by channel noises increased with the rising total degree of a parity-check matrix [12]. But they were not easily adjusted to the variable channels. So, G. Liva, et al. [7] suggested the encodable QC-based generalized LDPC codes. But the codes can not adequately utilize QC encoding structure and thus increased encoding complexity. So, an efficient code generation was proposed, splitting the base template matrices with the progressive edge growth (PEG) algorithm [13]. Additionally, one minimum QC sub-matrix in the protograph code matrix was eliminated to solve the rank deficiency problem without destroying the QC structure. By extending the approximate cycle extrinsic message degree (ACE) algorithm [14] in the QC-LDPC codes to find the circulant offsets in the QC sub-matrices [15], the protogrph QC-LDPC codes were designed to obtain improved performance compared to other codes of the same rate. Due to the QC structures, the codes also possessed low linear encoding complexity in proportion to the code length. The error floor was analyzed by asymptotic weight distributions (AWDs) [16]. And the low-rate codeword was designed with the technique of protograph extensions [17]. So, a low-error floor was achieved by increasing some repetitions of the information nodes in their Tanner graph to enlarge the minimum distance [18,19]. Moreover, they also obtained lower complexity and less latency in decoding [20]. And the construction of short and high-rate protograph QC-LDPC codes were presented with high-rate and rational complexity [21]. Due to the QC structures, the codes also possessed low linear encoding complexity in proportion to the code length. LDPC codes were dominant in satellite communication coding schemes based on the correlated reliability between the extrinsic information and the mapping processing information to improve the correctness of decoding [22]. And the multiplication in the iterative process was replaced by the simple sum of the Hamming distance coefficient to reduce the complexity and storage of the aeronautical telemetry system [23]. Furthermore, the random puncturing and shortening of the LDPC codes can further improve spectrum efficiency with the robust decoding for aeronautical telemetry purpose [24].

To construct efficient high-rate QC-LDPC codes, we focus on both the threshold and error floor, using the EXIT chart, PEG, and AWD techniques, for efficient QC-LDPC coding. We mainly consider a seed protograph-based code design, which facilitates the high-rate, encodable codes with both a good threshold and error floor. First, the Tanner graph is generated by the extension of a protograph with variable extensions using the EXIT chart. Second, the PEG and AWD schemes are also given to jointly optimize the specific code characteristic of the systematic construction of the proposed codes. Third, optimal circulant offset search of the QC sub-matrices is performed in QC-LDPC codes using the QC-NLACE algorithm. Finally, the main contributions are summarized as follows.

- Construction of the seed protographs with variable extensions by the EXIT chart.
  For the protograph QC-LDPC code, the edges in the optimized ARA protograph (as the seed protograph) are modified under the prediction of an EXIT chart resulting in a better threshold than that of the original protograph. We mainly modify this seed protograph by increasing or eliminating several connections in the graph to form a much more closed relationship of message propagation, thus reducing the threshold of the codes. Therefore, the EXIT charts are used to optimize the degree profiles to achieve a good decoding threshold and low error floor.
- Optimized seed QC matrix from the protograph using the PEG and asymptotic distance distribution techniques.
  The LDPC BP method obtained the optimal maximum likelihood decoding with a cycle-free Tanner graph of the code. But the cycle-free requirement is usually hard to achieve, due to the limited code length constraint. The short cycles cause performance deterioration due to the self-feedback of false messages in cycles. So, the codes are constructed with the largest possible girth under a finite code length. Followed by this criterion, the heuristic PEG algorithm is used to construct the code by adding every edge one by one, to guarantee the largest possible loop length. Then, decoding is improved by more accurate decision-making in BP decoding. In general, larger girth in the design of a QC-LDPC code leads to better code performance. A maximum average girth searching is utilized to optimize the structures of the QC-LDPC codes.
- Optimal circulant offset search of the QC sub-matrices by the QC-NLACE scheme.
  To obtain good QC-LDPC codes, the code with large girth, i.e., the smallest cycle, needs to be noticed. Since they cannot avoid all cycles, the relationships of the cycles are also crucial to the performance. Subsequently, the optimal code can be found by the ACE algorithm under the degree profiles and the QC framework. By extending the approximate cycle extrinsic message degree (ACE) algorithm [14] in the QC-LDPC codes, such as via the QC-NLACE algorithm, to find the circulant offsets in the QC sub-matrices, the QC-LDPC codes are designed to obtain improved performance compared to other codes of same rate. Therefore, the circulant offsets in the QC sub-matrices are searched by the improved QC-NLACE algorithm.

This paper is organized as follows. In Section 2, the protograph QC-LDPC codes are briefly introduced with their extensions. Then, in the next Section 3, the construction of the framework of the efficient high-rate and short-length QC-LDPC codes is also proposed, where a typical code graph is generated by an extension of a seed protograph with the variable node extension with the EXIT chart for optimized degree profiles. And the template matrix derived from the base Tanner graph is further divided and thus optimized by the PEG algorithm. In addition, the circulant offsets in the QC permutable sub-matrices are also searched by the QC-NLACE algorithm. In Section 4, the numerical simulations and the analyses of the QC-LDPC codes produced by the proposed scheme on an additive white Gaussian noise (AWGN) channel are given to verify good performance and the complexity is also quantitatively analyzed. Finally, the summary is drawn in Section 5.

## 2. Protograph QC-LDPC Codes

### 2.1. LDPC Codes with Their Check Matrices and Tanner Graphs

**Figure 1:**
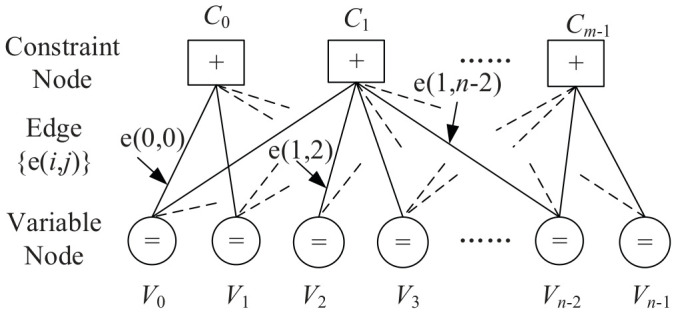
Tanner (bipartite) graph for a typical LDPC code.

LDPC code is mainly represented by an m×n low-density parity-check matrix **H**, which corresponds to a code with rate (n−m)/n, where *n*, *k*, and m=n−k are the code length, number of information bits per codeword, and parity-check bits per codeword, respectively. So, in the Tanner graph of the code, there are *m* check nodes, one for each row of **H**, and *n* variable nodes, one for each column of **H**. For the general case where **H** has irregular row and column weight, the Tanner graph can be characterized by the degree assignment sets ${\left\{d_{v}\left(i\right)\right\}}_{i=1}^{n}$ and ${\left\{d_{c}\left(j\right)\right\}}_{j=1}^{m}$, where $d_{v}\left(i\right)$ is the degree of the *i*-th variable node, and $d_{c}\left(j\right)$ is the degree of the *j*-th check node [5]. For an LDPC code, the variable and check degree profile, i.e., λ(x) and ρ(x), which influences the code performance, are depicted, respectively, as

(1) $$\lambda \left(x\right)=\sum_{i=2}^{d_{v}}\lambda_{i}x^{i-1},$$

(2) $$\rho \left(x\right)=\sum_{i=2}^{d_{c}}\rho_{i}x^{i-1},$$

where $d_{v}$ and $d_{c}$ are the maximum variable and check node degree. The degree profile is assumed as the number of the edges connected to the variable or check nodes, respectively, where $\lambda_{i}$ is the fraction of edges connected to variable nodes of degree *i*, while $\rho_{i}$ is related to check nodes of degree *i*. The degree profiles determine the theoretic decoding threshold of the codes, which can be searched by the density evolution technique [2]. So, the optimal Tanner graphs of the codes can be designed under the constraints of the degree profiles. With the notation of degree profile, a bipartite graph (protograph or Tanner graph) is the representative of the iterative decoder just as depicted in Figure 1. And each node represents a soft-in/soft-out processor (or node decoder).

In Figure 1, the variable nodes (VNs) are the cycle nodes with a sign “=” inside and marked by $V_{0},V_{1},\cdots ,V_{\left(n-1\right)}$ to indicate the *n* variable nodes, which comprise *k* information nodes and *m* parity-check nodes. The constraint nodes (CNs) are the square nodes with a sign “+” inside and marked by $C_{0},C_{1},\cdots ,C_{\left(m-1\right)}$ to signify the *m* constraints placed on the code bits associated with the CNs. The VNs connected with the same CN are involved in the same parity-check relationship. And the connection is depicted as the edge e(i,j), which builds a link between the CN *i* and VN *j*.

Since a bipartite graph gives a complete description of an LDPC code, the specifications of the component (linear) block are also required. By using the same terminologies in [5], we define $V={\left\{V_{j}\right\}}_{j=0}^{n-1}$ and $C={\left\{C_{i}\right\}}_{i=0}^{m-1}$ be the set of *n* variable nodes (VNs) and the set of *m* check nodes (CNs) in the bipartite graph of a QC-LDPC code, respectively. The connection between the CNs and VNs are summarized in a m×n base matrix **T**. Then, **T** is extended and structured as an adjacency matrix γ with quasi-cyclic structure, where each “1” and “0” in **T** being replaced with a cyclic sub-matrix Π[e(i,j)] (short for $\Pi_{i,j})$ and a zero matrix of the same dimension, respectively. And the cyclic sub-matrix is usually derived from circulant shift of an identity matrix. So the relationship between the adjacency matrix Γ and the parity-check matrix **H** for a QC LDPC code is determined, once each cyclic sub-matrix $\Pi_{i,j}$ is confirmed. In this case, Γ is an array of m×n circulant permutation matrices in the form of

(3) $$\Gamma =\left[\begin{array}{cccc} \Pi_{0,0} & \Pi_{0,1} & \cdots & \Pi_{0,n-1}\\ \Pi_{1,0} & \Pi_{1,1} & \cdots & \Pi_{1,n-1}\\ \vdots & \vdots & \vdots & \vdots \\ \Pi_{m-1,0} & \Pi_{m-1,1} & \cdots & \Pi_{m-1,n-1} \end{array}\right],$$

where each $\Pi_{i,j}$ is either a q×q circulant permutation matrix or a *q* × *q* full zero matrix. So the matrix Γ is just constructed by substituting circulant permutation matrices for the 1’s in the base matrix. The 0’s in Γ are replaced by all-zero q×q matrices. As a result, short cycles can be avoided with circulant permutation expansion. So, the final check matrix **H** is an optimized permutation case of the binary matrix Γ, with size of mq×nq, which is enlarged for *q* times of the base matrix in the dimension of both rows and cows. In summary, the check matrix **H** of a generalized QC-LDPC code can be obtained by the expansion of a base matrix **T** in the form of the adjacent matrix Γ with determined circulant permutation matrices, which are optimized for large cycles in their Tanner graphs.

### 2.2. The Multi-Edge Protographs with Extensions

A protograph is similar to the Tanner graph with a relatively small number of nodes, except that it can possess parallel edges, i.e., two nodes can be connected by more than one edge. It can be extended to a larger graph by a “copy-and-permute” procedure. This procedure includes firstly making *N* copies of the protograph and then permuting the nodes of each edge among the N variable and check nodes connected to the set of *N* edges copied from the same edge in the protograph.

During the “copy-and-permute” procedure, the connection relationship between the same sequence of the variable and constraint nodes must be maintained among different copies of the original protograph. The derived graph is the graph of a code N times of the code corresponding to the protograph, with the same rate and distribution of variable and check degrees. Decoding thresholds for infinite block size can be computed using density evolution or EXIT techniques on the protograph. For a given number of variable and check nodes in a protograph, all possible connections between variable and check nodes can be searched over to obtain a protograph with the lowest threshold. Some protographs of capacity approaching codes have been searched with good decoding threshold performance by extensive computer searching techniques. Compared with the repeat-accumulate (RA) code, the performance improvement of the ARA protograph code is mainly made by the pre-coding of the RA code with a series of differential encoders. So the performance can be improved by replacing the differential encoder with a more powerful Hamming encoder. Then, the codes are extended from the basic protograph-based code to a more powerful code. And the code can be implemented with the QC structure, which further reduces encoding and decoding complexity. So, the extensions provide great flexibility to construct codes with efficient encoding, good thresholds, and low floors.

## 3. Construction of Proposed QC-LDPC Codes

### 3.1. The Seed Protographs with Variable Extensions by the EXIT Chart

To construct a protograph code with a good threshold and low floor, the optimized design of the seed protograph, as well as the edge connections, is crucial. Among all LDPC codes with the protographs, the protographs of ARA codes can be the proper template for code extensions. They are characterized by a good threshold and simple representations. So we mainly discuss the protograph ARA-like QC-LDPC codes with simple edge modification to generate the high-rate codes (*R* > 1/2).

The EXIT chart technique is introduced in the design of protograph QC-LDPC codes in the AWGN channel model [14]. The additional metric updates of the punctured nodes in the EXIT chart are still emphasized. In the decoding, the variable node decoders (VNDs) and the check node decoders (CNDs) work iteratively to make decisions, with the metrics of interested bits improved with each half-iteration. A transfer curve of the input metrics versus the output metrics is obtained for both the VNDs and the CNDs, which depends on the channel SNR. In addition, because the output metrics for one decoder is just the input metrics for its companion decoder, both transfer curves can be plotted on the same figure with reversed axes. This chart can predict the decoding threshold of the ensemble of the codes featured by the given VN and CN degree profiles. The decoding threshold is the SNR where two transfer curves just encounter. So, the EXIT chart calculations of the decoding threshold are integral to the optimization of Tanner graph node degree profiles for the QC-LDPC protographs, which are the main optimization computation.

The predicted threshold is only the theoretic performance of a graph with no cycles and infinite code length and decoding iterations, as well as all assumed messages being processed in Gaussian distribution. But it is of great reference value for designing the proper protograph. The EXIT chart is used to analyze the theoretic threshold. But the threshold is only of function when the input/output metrics of the nodes follow the Gaussian distributions, and the code length is infinite for such threshold result. It is actually an unlimited performance bound for the codes with the protographs, and it is used to evaluate the construction method of the optimized codes. Since it is quite easy for calculation of the theoretic threshold of a seed protpgraph, it eases the analyses of the threshold of the codes, rather than the more complex prediction calculation from density evolution or Gaussian approximation.

**Figure 2:**
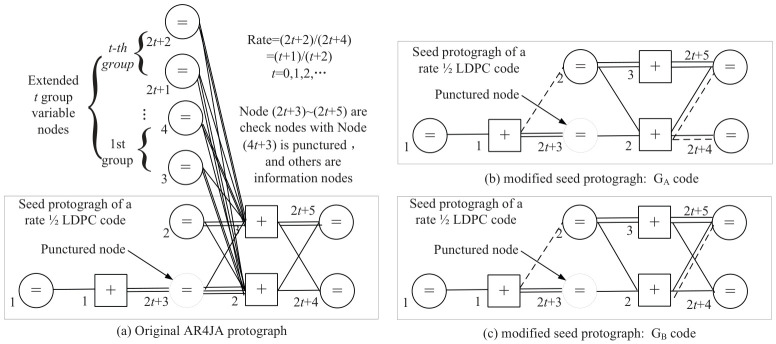
Protograph QC-LDPC codes with simple edge modification.

For this protograph QC-LDPC code, the edges in the optimized ARA protograph (as the seed protograph) are modified under the prediction of an EXIT chart for a better threshold than that of the original protograph. We mainly modify this seed protograph by increasing or eliminating several connections in the graph to form a much more closed relationship of message propagation, thus even increasing the randomness of the codes. The base protograph here is chosen from the famous AR4JA code family in CCSDS standard [20]. Some possible tentative edge changes are made as in Figure 2.

In Figure 2a, the original protograph is given with the seed protograph of a rate-1/2 AR4JA code [20]. Given the same extension of code rate by additional variable nodes in the top of Figure 2a, the modification mainly lies in the seed protograph. From Figure 2b,c, edges are modified as two main type code as $G_{A}$ and $G_{B}$ codes, respectively. Some of the modified edges are displayed in dashed lines pointed out in the seed protographs, where they are tentatively reversed and testified by the EXIT charts for good threshold. In practice, the code performances are affected by the code length and cycles in the Tanner graph and so on. Therefore, the pragmatic codes are designed with all of the above factors considered.

### 3.2. Optimized Base QC Matrix from the Protograph

**Figure 3:**
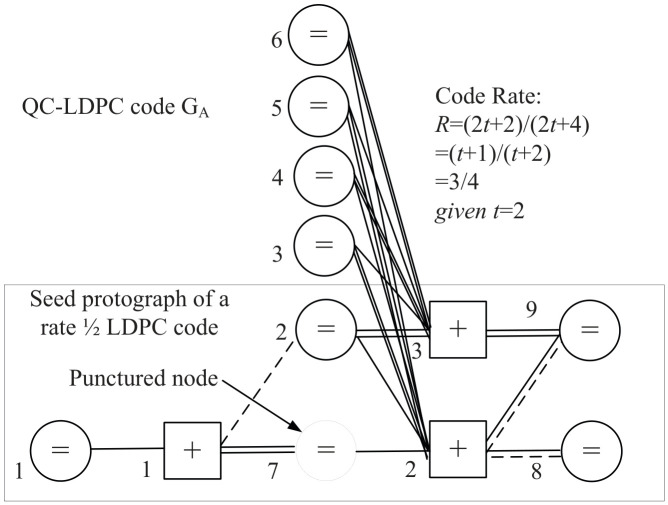
Typical rate 3/4 protograph QC-LDPC code $G_{A}$ with t=2.

Taking the protograph $G_{A}$ code of rate 2/3 (i.e., t=1) in Figure 2b, for example, we can get the new generated protograph as shown in Figure 3. The variable nodes from node-1 to node-6 are the information bit nodes, and those of node-8 and node-9 are the check bit nodes, while node-7 is the punctured node. The base matrix is then obtained in (4) according to the relationship of the protograph and parity-check matrix mentioned in Section 2.1.

(4) $$\left[\begin{array}{ccccccccc} 1 & 1 & 0 & 0 & 0 & 0 & 2 & 0 & 0\\ 0 & 1 & 2 & 1 & 2 & 1 & 1 & 2 & 2\\ 0 & 2 & 1 & 2 & 1 & 2 & 0 & 0 & 2 \end{array}\right].$$

The extended base code matrix **T** is then generated from (4) as (5), where all “1” and “2” are replaced with square circulant sub-matrix as $\left\{I_{1},I_{2}\right\}$, shifted from first row of weight-1 and weight-2, respectively, and all “0” are replaced with square zero matrix.

(5) $$T=\left[\begin{array}{ccccccccc} I_{1} & I_{1} & 0 & 0 & 0 & 0 & I_{2} & 0 & 0\\ 0 & I_{1} & I_{2} & I_{1} & I_{2} & I_{1} & I_{1} & I_{2} & I_{2}\\ 0 & I_{2} & I_{1} & I_{2} & I_{1} & I_{2} & 0 & 0 & I_{2} \end{array}\right].$$

In the code design, the extended code matrix **T** has to be further divided to improve the randomness of the matrix and thus enhance the code performance. In other words, the circular square sub-matrices $I_{1}$ and $I_{2}$ are needed to split and randomize the distribution of ‘1’ in the final check matrix. For instance, each square circulant matrix $I_{1}$ or $I_{2}$ in the base protograph is divided into a 4 × 4 square circulant matrix in (5), as long as it guarantees row and column weight in the original matrix **T**.

(6) $$I_{1}=\left[\begin{array}{cccc} I_{1}^{\prime } & 0 & 0 & 0\\ 0 & 0 & I_{2}^{\prime } & 0\\ 0 & I_{3}^{\prime } & 0 & 0\\ 0 & 0 & 0 & I_{4}^{\prime } \end{array}\right],I_{2}=\left[\begin{array}{cccc} I_{1,1}^{\prime \prime } & 0 & I_{1,3}^{\prime \prime } & 0\\ 0 & I_{2,2}^{\prime \prime } & 0 & I_{2,4}^{\prime \prime }\\ 0 & I_{3,2}^{\prime \prime } & I_{3,3}^{\prime \prime } & 0\\ I_{4,1}^{\prime \prime } & 0 & 0 & I_{4,4}^{\prime \prime } \end{array}\right]$$

In (6), $\left\{I_{i}^{\prime },I_{i,j}^{\prime \prime }\right\}$ are the embedded sub-matrices after the split of $\left\{I_{1},I_{2}\right\}$. The PEG algorithm [2] is then used to locate the optimal distribution of $\left\{I_{i}^{\prime },I_{i,j}^{\prime \prime }\right\}$ in the sub-matrix $\left\{I_{1},I_{2}\right\}$ of **T**. Accordingly, the detailed steps of the PEG algorithm is listed as follows.

**Figure 4:**
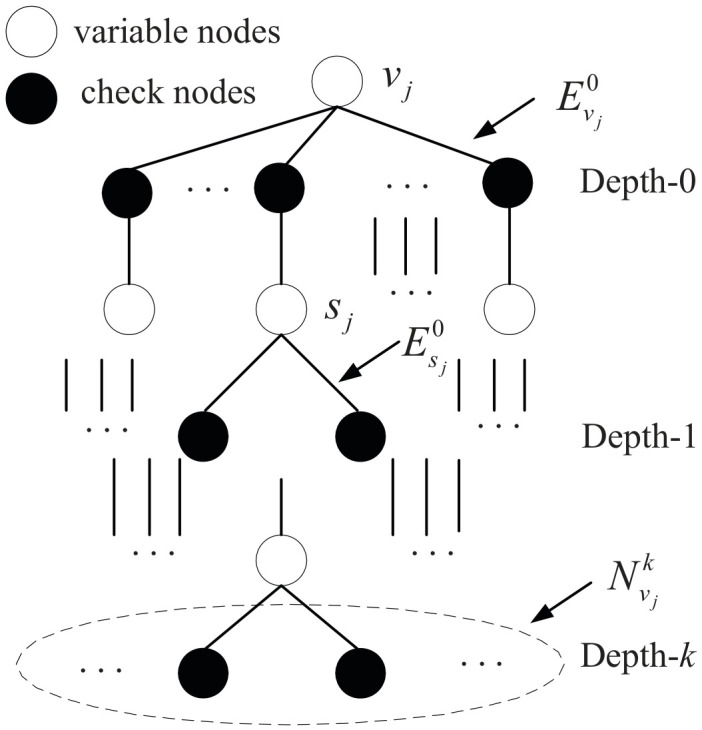
Tree structure of a Tanner graph expansion by the PEG algorithm.

The Tanner graph of the code can be reorganized as a tree structure, where the root node is chosen from an initial variable node. A typical tree with the root node, i.e., the variable node $v_{j}$, is shown in Figure 4.

In Figure 4, the layer of the check nodes away from the root node is called depth. $N_{v_{j}}^{k}$ is defined as the neighbor of the check node $v_{j}$ in depth-*k*, and $\bar{N_{v_{j}}^{k}}=V_{c}\setminus N_{v_{j}}^{k}$ is denoted as its complement, where $V_{c}$ represents the set of all check nodes in the Tanner graph. The distance between the first variable nodes (or check nodes) in depth-*k* and the root node is 2k and 2k+1, respectively. So, $N_{v_{j}}^{k}$ is also a set of all check nodes with distance (from the root node $v_{j}$) ≤ 2k + 1. Finally, $E_{s_{j}}^{0}$ is the first edge connected to the variable node $s_{j}$.

Given the above notations, the detailed procedure of the PEG tree search for the base Tanner graph of the code is expressed as follows.

Step (1). Given a variable node $s_{j}$, try to add the first edge, and then search out the check node $c_{i}$ with the minimum number of connected edges in the expanded sub-graph. Then, connect $s_{j}$ and $c_{i}$ as the first edge $E_{s_{j}}^{0}$ of node $s_{j}$.

Step (2). Place the remaining edges of the variable node $s_{j}$, and the sub-graph is thus expanded to a new layer with increased depth. Judge if ($\bar{N_{s_{j}}^{k}}\neq \phi$ and $\bar{N_{s_{j}}^{k+1}}\neq \phi$), or if the node number in set $N_{s_{j}}^{k}$ does not increase and it is less than the number of all check nodes, and then connect the variable and the check node in the set $\bar{N_{s_{j}}^{k}}$, where the number of edges connected to the check node is the minimum.

Step (3). Execute Step (1) and Step (2) repeatedly until all variable nodes are connected and thus the final Tanner graph is obtained.

By using the above PEG tree search Tanner graph generation method, the optimal distribution of $\left\{I_{i}^{\prime },I_{i,j}^{\prime \prime }\right\}$ in the sub-matrix $\left\{I_{1},I_{2}\right\}$ of **T** is confirmed. In addition, the sub-matrix $\left\{I_{1},I_{2}\right\}$ can also be divided in a higher dimensional matrix with 8 × 8 or 16 × 16, and so on, to further improve the code randomness for better performance. However, it increase the number of the circulant vectors and thus also increase the storage of their positions too. So, the performance and the complexity (refer to resource) should be compromised for overall performance. Then, a typical LDPC code with code length 1024 (due to the puncture, the appearance code length is 1280) and rate 3/4 is constructed by 4 × 4 division of each 128 × 128 square circulant matrices in matrix **T**. Finally, a 4 × 4 split sample matrix, i.e., the base matrix for the final check matrix **H**, is shown in (7).

(7) $$\left[\begin{array}{ccccccc} \cdots & 0 & 0 & 0 & 0 & \begin{array}{cccc} I_{1,1}^{\prime \prime } & 0 & I_{1,3}^{\prime \prime } & 0\\ 0 & I_{2,2}^{\prime \prime } & 0 & I_{2,4}^{\prime \prime }\\ 0 & I_{3,2}^{\prime \prime } & I_{3,3}^{\prime \prime } & 0\\ I_{4,1}^{\prime \prime } & 0 & 0 & I_{4,4}^{\prime \prime } \end{array} & \cdots \\ \cdots & \vdots & \vdots & \vdots & \vdots & \vdots & \cdots \end{array}\right].$$

The circulant matrices are assigned randomly only if the entire QC matrix is full rank. However, the code designed in [5] cannot be implemented in simple QC form, since it easily has problem of rank deficiency in the matrix derived from the protograph, where the module-2 addition of all rows in first two block row of matrix **T** together must be the zero vectors. So, we use the equivalent technique of truncation as in Turbo decoding, by eliminating a smallest circulant sub-sub-matrix in first two block row of matrix **T** to achieve full rank. In our practice, we eliminate a smallest circulant sub-sub-matrix in the position **T**(3,2) of (5). Therefore, we can generate the code in the QC form of full rank, which simplifies the encoding, since the code with full rank QC matrix is much more regular and easy to be implemented. In addition, the circulant offsets in the sub-matrices are searched optimally in the next subsection with the QC-NLACE algorithm to obtain good decoding threshold and low error floor.

### 3.3. Minimum Distance Improvement of the LDPC Codes with the AWD

Given code length *n*, rate *R*, and Hamming distance *d*, the average normalized code weight of the variable node for transmission is δ=d/n. Suppose that $A_{\delta }$ is the weight distribution of the code ensemble, i.e., the number of the codeword with weight *d*, and the normalized AWD function [24] is presented as

(8) $$r\left(\delta \right)=\lim_{n\to \infty }\sup[\ln\left(A_{d}\right)/n=\lim_{n\to \infty }\sup\left[\ln\left(A_{\delta n}\right)/n\right].$$

For an LDPC code, if there is cross-zero point in r(δ), and $r\left(\delta \right)<0,\left(0<\delta <\delta_{\min}\right)$ for the first cross-zero point $\delta_{\min}>0$, $\delta_{\min}$ is set as typical minimum distance ratio (TMDR). At this moment, the Hamming distance *d* meets the unequal equation $d>\delta_{\min}\cdot n$, namely, there is

(9) $$\forall \varepsilon >0,P(d>\delta_{\min}n)>1-\varepsilon .$$

Thus, the approximate minimum distance is obtained as

(10) $$d_{\min}\approx \delta_{\min}\cdot n.$$

For a random code, the AWD function is expressed as

(11) r(δ)=(R−1)ln2+H(δ),

where H(δ) is the entropy function and is given by

(12) H(x)=−xlnx−(1−x)ln(1−x).

For an LDPC code, the function of the asymptotic weight distribution [18] is thus calculated as follows. Suppose the base matrix B for an LDPC code is presented as the matrix element set of ($b_{ij}$), the variable node set of the code is $S_{t}\left(\right|S_{t}\left|=n_{t}\right)$, and the number of the transmitted nodes is $n_{t}$. The number of the punctured nodes is $n-n_{t}$. The normalized code weight and the degree of each variable node $v_{i}$ are $x_{j}$ and $q_{j}^{v}$, respectively. In addition, the degree for every check node $q_{i}^{c}$ is 3. Then, r(δ) is represented as

(13) $$r\left(\delta \right)=\max_{x_{j}\in X}\left\{\sum_{i=1}^{n_{c}}a^{c_{i}}\left(x_{j1},x_{j2},x_{j3}\right)-\sum_{j=1}^{n_{v}}\left(q_{j}^{v}-1\right)H\left(x_{j}\right)\right\}/n_{t},$$

where some parameters are expressed, respectively, as

(14) $$a^{c_{i}}\left(x_{j1},x_{j2},x_{j3}\right)=H_{4}\left(\sigma -x_{j1},\sigma -x_{j2},\sigma -x_{j3},1-\sigma \right),$$

(15) $$\sigma =(x_{j1}+x_{j2}+x_{j3})/3,$$

(16) $$H_{4}\left(x_{1},x_{2},x_{3},x_{4}\right)=-\sum_{j=1}^{4}x_{j}.$$

subject to the constraint of

(17) $$\left\{\begin{array}{c} 0\leq x_{j}\leq 1\\ \max\{x_{j1},x_{j2},x_{j3}\}<\sigma <1\\ \sum_{x_{j}\in s_{t}}x_{j}=n_{t}\delta ,0\leq \delta \leq 1 \end{array}\right.,$$

where {$x_{j1}$,$x_{j2}$,$x_{j3}$} are the normalized weight of the 3 variable nodes associated with the same check node $c_{i}$.

**Figure 5:**
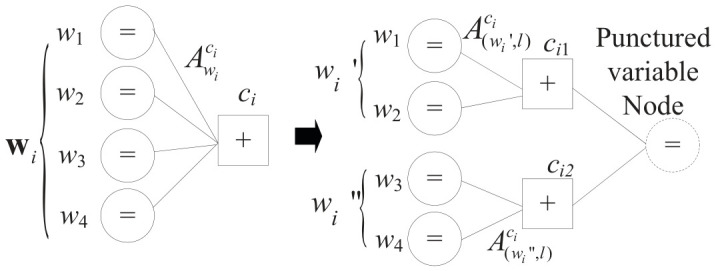
Check node with degree-4 and its equivalent representation.

Since all check nodes are required to be the same degree 3 in this algorithm, it must use the spitting algorithm to process the check node without degree-2 or degree-3 [24]. The degree $q_{i}^{c}$ of a check node larger than 3 must be spitted as $q_{i}^{c}-2$ sub-check nodes with degree-3 and $q_{i}^{c}-3$ punctured variable nodes with degree-2. A typical spitting of check node with degree-4 is shown in Figure 5. The weight enumerator $A_{w}^{c_{i}}$ of the check node $c_{i}$ for this protograph code ensemble is achieved by accumulating contributions from partial weight vectors $w_{i}$ ($w_{k},k=1,\cdots ,4$). The partial weight vectors $w_{i}$ is then divided by two degree-2 partial weight vectors $w_{i}^{\prime }$ and $w_{i}^{\prime \prime }$ for calculating $A_{\left(w_{i}^{\prime },l\right)}^{c_{i}}$ and $A_{\left(w_{i}^{\prime \prime },l\right)}^{c_{i}}$. The partial weight vectors of the two new checks are denoted as ($w^{\prime },l$) and ($w^{\prime \prime },l$), where $w^{\prime }$ and $w^{\prime \prime }$ are subvectors of the original node’s partial weight vector $w_{i}$, and *l* is the weight associated with the degree-2 punctured variable node. Finally, they are combined by a punctured variable node for equivalent AWD calculation.

Finally, the function of the asymptotic weight distribution is used to analyze the floor performance of the LDPC code in the high-SNR region. Thus, it helps to design the codes with a low error floor. Given the code length *n*, a larger $\delta_{\min}$ leads to a larger minimum distance, and thus the lower floor. Also it can be used to calculate the threshold $\gamma_{\mathrm{ml}}$ of the maximum likelihood decoding as    

(18) $$\gamma_{\mathrm{ml}}=\frac{1}{2R}\underset{\delta }{\cdot \max}\left[1-e^{-2r(\delta )}\right]\cdot \left(1-\delta \right)/\delta ,$$

where *R* is the code rate.

### 3.4. Optimal Circulant Offset Search by the QC-NLACE Algorithm

To obtain good QC-LDPC codes, the code with large girth, i.e., the smallest cycle length, needs to be noticed. Since they cannot avoid all cycles, the relationships of the cycles are also crucial to the performance [10]. Then, the optimal codes are found by the QC-NLACE algorithm under the degree profiles and the QC framework. Or the circulant offsets in the QC sub-matrices are searched by the proposed QC-NLACE algorithm, and it is presented as follows.

The QC-NLACE algorithm is described in Algorithm 1. The optimal circulant offsets of the non-zero sub-matrix $\Pi_{i,j}$ in (3) are found by the ACE algorithm, and the parameters ($d_{ACE}$; $\eta_{ACE}$) are optimally selected by multiple tentative experiments with accurate ACE values under nested loops.

(1) Some initialized notations.

Notation 1 (EMD) [14]: An extrinsic constraint node of a variable node set is a constraint node singly connected to the set. The EMD of a variable node set is the number of extrinsic constraint nodes of this set.

Notation 2 [Approximate Cycle EMD (ACE)]: The ACE of a length 2d cycle is $\sum_{i}(d_{i}-2)$, where $d_{i}$ is the degree of the *i*-th variable node in the cycle. The ACE of a degree-d variable node is (d−2), and the ACE of any constraint node is 0.

Other notations: m×n sub-matrices of the circulant offset are searched. For all variable and check nodes, $p(\mu_{t})$ is the ACE between the node $\mu_{t}$ and the root node $v_{0}$ in the tree search. When $\mu_{t}$ is a variable node, ACE ($\mu_{t}$) is the degree of $\mu_{t-2}$. Otherwise ACE ($\mu_{t}$) is 0.
**Algorithm 1** The flow chart of the QC-NLACE algorithm**Initializations**: m×n sub-matrices for searching the QC offsets are required. For all variable and check nodes, $p(\mu_{t})$ is the *ACE* between any node $\mu_{t}$ and the root node $v_{0}$ in the tree search of the code. When $\mu_{t}$ is a variable node, $ACE(\mu_{t})$ is the degree of $\mu_{t}$ minus 2. Otherwise $ACE(\mu_{t})$ is 0.1:**for** (*i* = *n*− 1; *i*≤ 0; *i*− −)2:   **begin**3:   **for** (*j* = 0; j≤m− 1; *j*++)4:      **begin**5:      **redo:**6:      Randomly generate $v_{i}$ according to the non-zero offset positioning in the first row of the QC sub-matrix Γ which is not a zero matrix.7:      ***ACE*** **detection of $v_{i}$:**          $p(\mu_{t})\leftarrow \infty$ for all variable and check nodes.          $p\left(v_{i}\right)\leftarrow ACE\left(v_{i}\right)$ for all variable and check nodes.          **Find all loops in current topology.**          Given one variable node as the root, track the connected edges from it to any possible nodes by the depth-first-search graph algorithms and record the node set of a loop. If there are different node loop sets with the same root node, the sets belong to the same nested loop, otherwise they are single loops. Nodes in a nested loop are marked for successive procedure.          **For signle loops:** $ACE\left(v_{i}\right)=d_{i}-2$. $ACE(c_{i})=0$. The ACE of a length 2d loop is $\sum_{i}(d_{i}-2)$. $v_{i}$ and $c_{i}$ are the *i*-th variable and check nodes, respectively.          **For nested loops:** Suppose the check node set of the nested loop is $C(v_{i})$, where all check nodes are connected to the same variable node $v_{i}$ in the common edges of the nested loops. The common variable nodes of all the nest loops are selected for more outer connections to obtain outer independent extrinsic information to avoid error self-propagation among loops. The number of $C(v_{i})$ is represented as $N(C\left(v_{i}\right))$. So, the ACE of the nested loop is calculated as $\sum_{i}\left(d_{i}-2\right)-\sum_{k}\left[N\left(C\left(v_{k}\right)\right)-2\right]$.8:      **for** (*k* = 1; $k\leq d_{ACE}$; *k*++)9:        **begin**10:        **for** any active node $w_{s}$ in level (k−1).11:         **begin**12:         Find its children set $Ch(w_{s})$.13:         **for** every child $\mu_{t}\in Ch\left(w_{s}\right)$.14:            **begin**15:            $p_{temp}\leftarrow p\left(w_{s}\right)+ACE\left(\mu_{t}\right)$.16:            $s_{temp}=p_{temp}+p\left(\mu_{t}\right)-ACE\left(v_{i}\right)-ACE\left(\mu_{t}\right).$17:            **if** ($s_{temp}<\eta_{ACE})$18:                 **Exit with failure.**19:            **else if** ($p_{temp}\geq p\left(\mu_{t}\right)$)20:                 Deactivate $\mu_{t}$ in level-*k* with current parent $w_{s}$.21:            **else**22:                 $p\left(\mu_{t}\right)\leftarrow p_{temp}$23:            **end**24:            **end**25:         **end**26:         **Exit with success.**27:         **if** ($ACE<\eta_{ACE}$ for a cycle of length 2$d_{ACE}$ or less)28:           **goto redo.**29:         **end**30:     **end**31:   **end**32: **end**

(2) The QC-NLACE algorithm is designed for the QC adjacent matrix searching and it is shown as follows.

Finally, the possible combinations can be tentatively search by the QC-NLACE algorithm, and the ($d_{ACE}$, $\eta_{ACE}$) is selected as (6,3) by multiple tries which are optimized for the short and high-rate code with the ACE criterion.

### 3.5. Joint Optimized LDPC Code Design

According to the above code design, the asymptotic weight distribution analysis, and the QC-NLACE technique, a joint optimized LDPC code design method is proposed for a good threshold and low floor. It compromises the performance between the threshold (waterfall region) and the error floor. Namely, it improves the error floor as well as the waterfall region. By the principle of protograph, an optimization of a small scale of protograph template leads to the final good performance for the code, which greatly reduces the construction complexity. So, an optimized protograph template of a rate 1/2 ARA code can be the proper seed protograph, which is extended to a high code rate by increasing variable nodes. Then, a base matrix Γ with an m×n circulant matrices can be designed under the parameters as an iterative decoding threshold γ, i.e., ${\left(E_{b}/N_{0}\right)}^{*}$, check node degree (prior: $q_{i}^{c}$ and posterior: $d_{i}^{c}$), the minimum distance ratio ($\delta_{\min}$), and the ACE parameter pair ($d_{ACE}$, $\eta_{ACE}$). And the procedure of the joint code design is listed as follows.

Step (1). Consider a m×n base matrix Γ, where $b_{ij}$ is the number of the edges between the check node (CN) $c_{i}$ and variable node (VN) $v_{i}$. Suppose the pre-coding flag is $l_{p}$, and $l_{p}$ is equal to 1 when there is the pre-coding structure in the protograph corresponding to matrix Γ. And the first row and cow of matrix Γ is fixed to keep the pre-coding gain. Namely, the edge between CN $c_{1}$ and VN $v_{1}$ is fixed. The QC ACE parameter pair ($d_{ACE}$, $\eta_{ACE}$) is also initialized here according to a similar coding experience of the same code length. Then, if $l_{p}$ is equal to 1, go to step (2), otherwise go to step (3).

Step (2). Set the degree of CN $c_{2}$ and $c_{3}$, i.e., $d_{2}^{c}$ and $d_{3}^{c}$, in Γ, as $q_{2}^{c}$ and $q_{3}^{c}$, or $q_{2}^{c}+1$ and $q_{3}^{c}-1$, respectively, where the degree of every check node is kept unchanged. Then, randomly choose the value of $b_{ij}$ to get $\Gamma_{1}$, on the condition that $\sum_{j=1}^{n}b_{ij}=d_{i}^{c}$. Subsequently, go to step (4).

Step (3). Set $d_{1}^{c}=q_{1}^{c}$, $d_{2}^{c}=q_{2}^{c}$, and $d_{3}^{c}=q_{3}^{c}$. Choose $b_{ij}$ according to the method in step (2) and get $\Gamma_{2}$. Then go to step (4).

Step (4). Calculate the threshold $\gamma_{2}$ of the matrix $\Gamma_{2}$. If $|\gamma_{2}-\gamma_{1}|<k$, go to Step (5), else if $l_{p}=1$, go to Step (2), and else if $l_{p}=0$, go to Step (3).

Step (5). Calculate the minimum distance ratio $\delta_{\min\;2}$ of the matrix $\Gamma_{2}$. If $\delta_{\min\;2}>\delta_{\min\;1}$ or $\delta_{\min\;2}$ does not exist, simulate the performance of $\Gamma_{2}$ by the EXIT chart and go to Step (6). Otherwise, if $l_{p}=1$, go to Step (2), else if $l_{p}=0$, go to Step (3).

Step (6). Given the tentative QC ACE parameter pair ($d_{ACE}$; $\eta_{ACE}$), calculate the circulant offsets for every sub QC matrix. When ($d_{ACE}$, $\eta_{ACE}$) is not satisfied, go to Step (1) with a degraded QC ACE parameter pair and undergo the above steps, until all circulant offsets are searched and satisfied for the QC ACE parameters. Then, the joint algorithm is over and the codeword is sent for the final BP decoding.

In the above process, *k* is a factor to adjust the threshold, which is a positive constant. If it is large, it is easily to find the code with a better threshold (waterfall region) than that of the original code. However, it usually has a worse error floor, which in turn increases the complexity of the iterative search computation. To the other extreme, a small *k* also leads to a poor threshold as well as a worse error floor. Thus, *k* should be compromised for floor and threshold, which is obtained as 0.4 dB and so on, by numerical simulations.

The proposed code design scheme is mainly composed of the seed protograph construction with variable extensions by the EXIT chart, the optimized seed QC matrix by the PEG and AWD, and the optimal circulant offset search of the QC sub-matrices by the QC-NLACE scheme. Then, the computational complexity of the proposed code design method can be the weighted combination of the above three methods by the required iterations. Therefore, the proposed code design scheme achieves a good threshold and error floor performance at the cost of the above-mentioned huge complexity.

Therefore, the whole code design procedure is implemented by the joint procedures of the seed LDPC protograph design, the QC matrix with the PEG and the AWD, and the circulant offset search of the QC sub-matrices by the QC-NLACE. And they are performed alternately to obtain the optimized LDPC code words with a good decoding threshold and error performance.

## 4. Simulation Results and Analysis

To verify the QC-LDPC codes by the proposed method, the BER of the codes are simulated and compared with the CCSDS AR4JA codes as well as the currently well-designed QC-LDPC codes and some other structured LDPC codes, respectively. And the decoding complexity for the designed code is also analyzed in practice. The results are as follows.

### 4.1. BER Simulations and Comparison of the Proposed Codes

#### 4.1.1. Comparison with Traditional LDPC Codes

**Figure 6:**
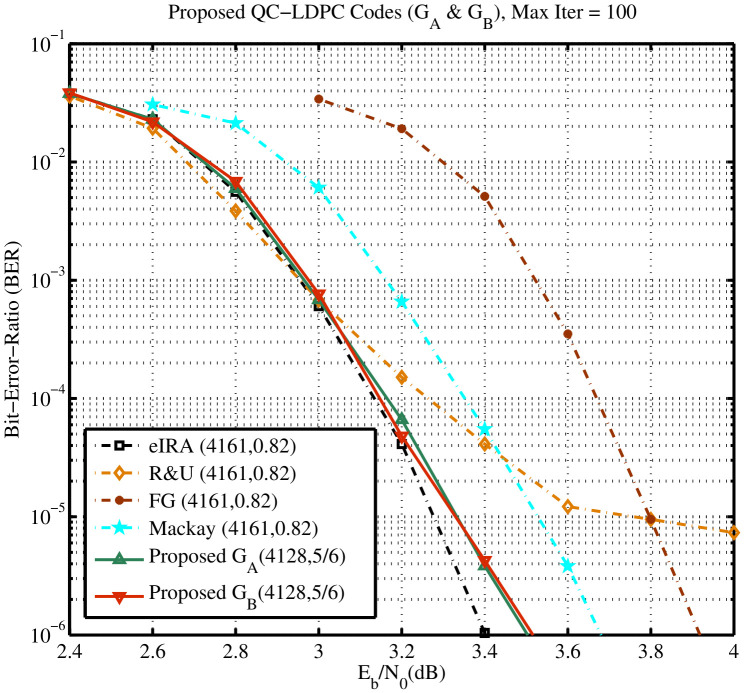
BER of the proposed $G_{A}$ & $G_{B}$ codes vs. traditional LDPC codes.

The simulation parameters are listed as follows. The proposed high-rate QC-LDPC codes, i.e., $G_{A}$ & $G_{B}$, with code length and information bit length (4128; 3440) are designed with the above method with code length 5/6 (approximate 0.8333). In contrasting schemes, the random or semi-structured extended irregular-repeat-accumulate (eIRA), R&U [3], finite geometry (FG) [25], Mackay codes [2] with the similar code length and rate (4161, 0.82) are used. With the above parameters, the BER for all codes are shown in Figure 6.

In Figure 6, the proposed QC-LDPC code obtains a little better performance over the contrast R&U [3], finite geometry (FG), and Mackay codes by 0.2–0.45 dB at BER of $10^{-5}$ on an AWGN channel. But, the proposed codes still have a slight performance loss when compared with that of the eIRA codes, though the latter has been optimally constructed with the semi-constructed code matrix. At low SNRs, the $G_{A}$ code outperforms the $G_{B}$ code slightly, because $G_{A}$ code has high variable weight at the node 2t+5 at the top right of Figure 2b. They also exhibit a slightly poor error floor for the codes. At high SNRs, the performance degrades a little. The proposed method adequately utilizes the seed QC matrix, PEG, AWD, and QC-NLACE to jointly improve both the threshold and error floor. However, existing code generation schemes are usually not used to optimize all possible joint code parameters to enhance code performance. The proposed method even uses the QC-NLACE to overcome the inevitable loop problem for even better error floor performance.

Compared with the random Mackay code, the R&U code, and the finite geometry code, the proposed codes ($G_{A}$ & $G_{B}$) have a better decode threshold at a higher bit error rate (larger than $10^{-5}$). It fully reflects the construction advantage of the optimized protograph template and thus reduces the difficulty of codeword construction. And it improves the efficiency of codeword construction. Compared with the semi-random eIRA code with optimal degree distribution, this code does not have the maximum degree of freedom of the random encoding matrix, and the degree distribution is far from the optimal distribution. So, it is still acceptable to have a trivial performance gap. And a full-structured quasi-cyclic code structure is adopted, so that the complexity of the code and decoding information storage and implementation is greatly reduced, which endows the ease of practical application for the proposed codes.

In addition, the code constructed by the proposed method adopts the measures of searching the cycle offset of the better ACE index and the measurement, which can eliminate short loops and improve the relation among them. It effectively overcomes the defect that the traditional QC-LDPC codes easily generate an error code floor. So, it better improves the comprehensive performance of the code. Especially in the construction of short code, due to the limitation of code length, the Tanner graph corresponding to LDPC coding is prone to unavoidable short loops, which makes its girth shorter. It leads to the self-feedback iteration of the error codeword message in the LDPC iterative decoding, and it causes the error propagation phenomenon and directly leads to the deterioration of decoding performance. The code word constructed by the code introduces the message passing edge of the external independent variable node as much as possible on the basis of the unavoidable compound loop, so that when the decoding message loop is wrong, the error can be corrected by the external independent correct message in time, thereby greatly reducing the error propagation and improving the decoding performance.

#### 4.1.2. Comparison with the CCSDS AR4JA Codes

**Figure 7:**
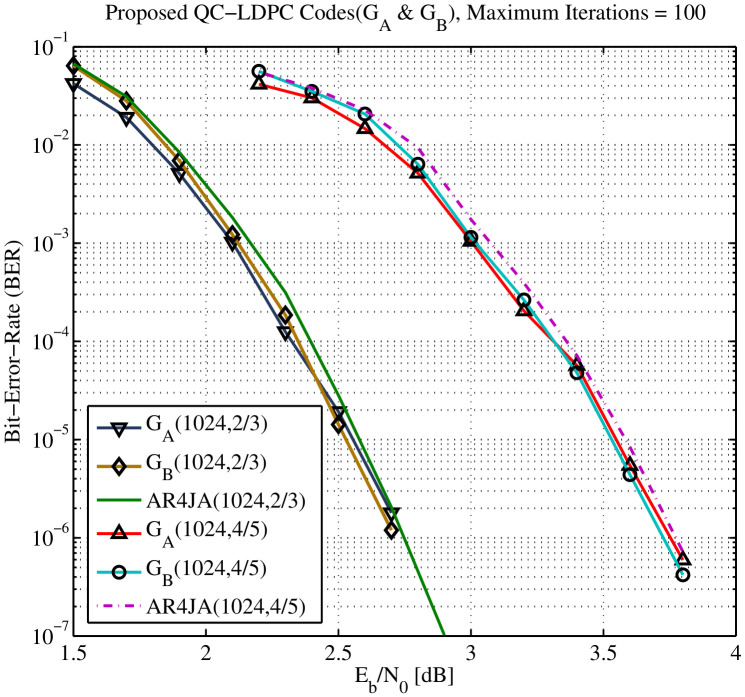
BER of the proposed $G_{A}$ & $G_{B}$ codes vs. AR4JA codes.

The simulation parameters are listed as follows. Short, high-rate QC-LDPC codes, i.e., $G_{A}$ & $G_{B}$, with information bit length and rate (1024, 2/3), (1024, 4/5) are designed with the above method. In contrast schemes, the CCSDS AR4JA codes [26] with the same code length and rate are adopted. With the above parameters, the BER for all codes are shown in Figure 7.

In Figure 7, the proposed QC-LDPC code obtains a slightly better performance over the contrast AR4JA codes by 0.03–0.05 dB at a BER of $10^{-5}$ on an AWGN channel. Our codes still have slight performance gains, though the AR4JA codes have been optimally constructed with the similar base protograph. At low SNRs, the $G_{A}$ code outperforms the $G_{B}$ code, because $G_{A}$ code has high variable weight at the node 2t+5, as seen in the top right of Figure 2b. But it also brings a little poor error floor for the code. So, at high SNRs, the performance degrades.

In this simulation, the proposed code still has a better decoding threshold and also reflects the construction advantage of the optimal expansion of the protograph template. In other words, the protograph template node has a lower decoding threshold after expansion. Because the AR4JA code also adopts a code with a template structure similar to a protograph and it is sufficiently optimized in the aspect of graph optimization construction, the code word constructed by the proposed method has little advantage relative to the AR4JA code. On the premise of similar implementation complexity, the code construction method obtains a slight performance improvement by adopting improvements such as expanding and optimizing check nodes and the similar techniques, thus it still has excellent practical significance.

#### 4.1.3. Comparison with Currently High-Rate QC-LDPC Codes

**Figure 8:**
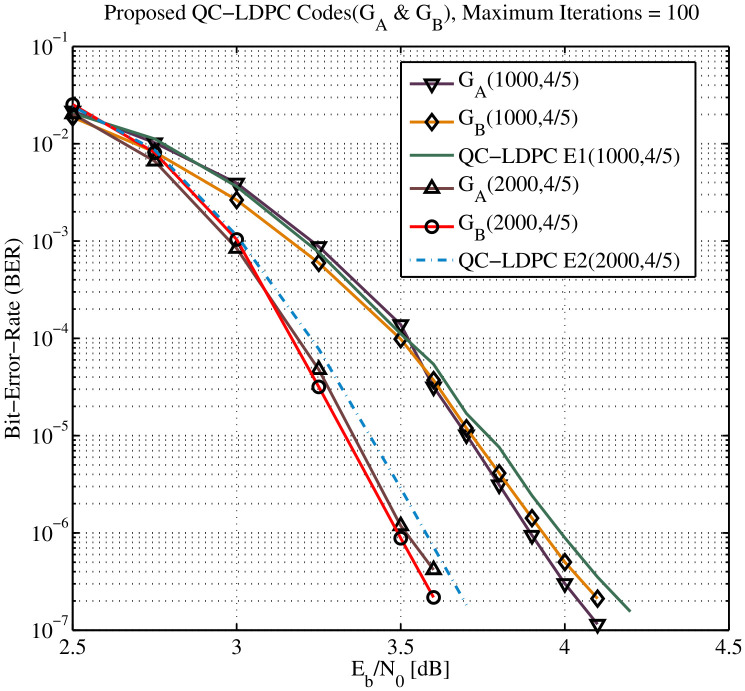
BER of the proposed codes vs. current QC-LDPC codes.

The simulation parameters are set as follows. The proposed codes of $G_{A}$ and $G_{B}$, with code length and rate (1000, 4/5), (2000, 4/5) are designed with the proposed scheme. The contrast QC-LDPC codes of E1 and E2 [27] with the same code length and rate are adopted. With above parameters, the BER for these codes are shown in Figure 8.

In Figure 8, the proposed QC-LDPC code obtains slightly better performance over the contrast QC-LDPC codes by 0.1–0.05 dB at a BER of $10^{-6}$ on an AWGN channel. The designs of the contrast codes concentrate the girth of the code, which neglects the relationships among the loops. Moreover, the ACE properties have not been improved for a better threshold and floor; thus, it still losses some performance when compared with those of the codes designed by the proposed code construction method.

In these scheme, the code length of the constructed codeword is not long enough, and the code rate is high. So, a large number of loops remain in the Tanner graph, and some loops are nested with each other. In other words, the partial edges of a loop are still the partial edges of another loop, resulting in the existence of a large number of common edges between rings. This easily leads to error messages that cannot effectively be removed as constraints from the self-feedback transmission between loops.

### 4.2. The Complexity Analyses of the Proposed QC-LDPC Codes

Pragmatic LDPC codes mainly suffer from high encoding complexity, which is in proportion to the square of code length. But in the QC form, the linear encoding complexity is in proportion to code length. It uses a linear feedback shift register to store the generator matrix, which is very well-tailored for implementation by resource-limited hardware.

On the premise of the same maximum variable, check node degree, and code length parameter *n*, the LDPC code has a linear encoding complexity of O(*n*). Meanwhile, the encoding complexity of all random LDPC codes is O($n^{2}$). The QC structure proposed in this paper makes full use of the quasi-cyclic structure, which only needs to store the first row matrix elements of the cyclic submatrix. Then, through the cyclic shift method, it can be used to realize the binary multiplication of the encoding matrix. Therefore, given the size of the circulant submatrix as p×p, the storage capacity is reduced to 1/p of the random matrix. So, a large number of storage units are saved, and the method is suitable for hardware implementation. In the premise of the same maximum variable, the same check degree, the same code length parameter *n*, and so on, due to the quasi-cyclic characteristic of a check matrix, the storage capacity of the decoding node position of the QC-LDPC code is 1/p of that of an original random check matrix. Thus, only the first row of the cyclic submatrix needs to be stored in a single cyclic position, and then p−1 positions are stored, and the relevant decoding node is completed by cyclic shifting, thereby reducing the necessary calculation and storage capacity. However, this codeword also has bears the cost of increasing the decoding complexity. When constructing the check matrix, it is realized by deleting part of the check columns. During decoding, the deleted check column needs to be added again for the decoding check. Given the ratio of the deleted column length to the code length as *r* and the column weight of the deleted column as *k* times of the average column weight before deletion, the decoding complexity is increased by about r×k times. The benefit is the greatly improved performance, which is slightly higher than that of the unconstrained fully random codewords. Moreover, the storage of the check position is reduced to 1/p of the original.

LDPC decoding can be realized with a parallel BP algorithm for low complexity. The QC structure also makes the decoding more efficient, since finding the position of the variable/check nodes in the memory is easier, saving considerable memory. Given appearance code length *n*, information bit length *k*, and check bit length *m* (i.e., n−k), average row and column weight as $w_{r}$ and $w_{c}$, iteration number *l*, the decoding (BP algorithm) complexity of the proposed code is listed in Table 1.

In practice, LDPC decoders are implemented in partially parallel manner given limited resources. So, the proposed method is flexible and easily adjusted to generate other high-rate QC-LDPC codes with efficient coding by selecting a proper seed protograph and some variable nodes for rate extensions.

## 5. Conclusions

In this paper, we propose an optimized construction of efficient, high-rate QC-LDPC codes. The Tanner graph is generated by the extension of a protograph template with variable extensions by the EXIT technique. Meanwhile, the PEG, AWD, and QC-NLACE techniques are also given to optimize the specific code characteristics in the systematic code construction for a good threshold and low floor. Simulation results show that the generated codes achieve good performance, by using the iterative BP decoding. In addition, the complexity of both encoding and decoding is suited for practical implementation. Therefore, the proposed code construction can be efficiently applied in coding practice with good performance and low complexity for the future 5G wireless communication applications.

## Figures and Tables

**Table 1:** Decoding complexity of the proposed code.

Additions	Module-2 (Xor)	Lookup Table
$\left(2\cdot l\cdot w_{c}-w_{c}+l\right)n+l\cdot m\left(w_{r}-1\right)$	$l\cdot m(w_{r}-1)$	$l\cdot m\cdot w_{r}$

## Data Availability

The data presented in this study are available on request from the corresponding author.

## Abbreviations

The following abbreviations are used in this manuscript

ARA	Accumulate-repeat-accumulate
AWGN	Additive white Gaussian noise
ACE	Approximated cycle extrinsic message degree
AWD	Asymptotic weight distribution
BER	Bit-error-rate
BP	Belief propagation
eIRA	Extended irregular-repeat-accumulate
EXIT	Extrinsic information transfer
LDPC	Low-density parity-check
PEG	Progressive-edge-growth
QC	Quasi-cyclic
QC-NLACE	Quasi-cyclic oriented nested loop approximated cycle extrinsic message degree
QC-LDPC	Quasi-cyclic low-density parity-check
SNR	Signal-to-noise ratio
